# Association between flat variants of the peroneus brevis tendon and split tears on magnetic resonance imaging

**DOI:** 10.1007/s00256-025-05032-y

**Published:** 2025-09-13

**Authors:** Rafał Zych, Dan Mocanu, Ymer Hagberg, Katarzyna Bokwa-Dąbrowska, Dawid Dziedzic, Katarina Nilsson Helander, Pawel Szaro

**Affiliations:** 1 Department of Clinical and Descriptive Anatomy, Medical University of Warsaw, Warsaw, Poland; 2 Department of Musculoskeletal Radiology, Sahlgrenska University Hospital, Gothenburg, Sweden; 3 Department of Radiology, Institute of Clinical Sciences, Sahlgrenska Academy, University of Gothenburg, Göteborgsvägen 31, 431 80 Gothenburg, Sweden; 4 Department of Orthopedics, The Institute of Clinical Sciences at Sahlgrenska Academy, University of Gothenburg, Gothenburg, Sweden

**Keywords:** Peroneus brevis tendon, Split tear, Magnetic resonance imaging, Tendon shape, Cross-sectional area, Ankle MRI

## Abstract

**Objectives:**

To determine whether the peroneus brevis tendon shape, cross-sectional area, and patient age are associated with split tears on magnetic resonance imaging.

**Methods:**

This retrospective cross-sectional study included 358 patients (179 with and 179 without split tears), with sample size based on an a priori power calculation (Cramér’s *V* = 0.186, 80% power, *α* = 0.05). Musculoskeletal radiologists assigned patients to split tear or no-tear groups based on MRI findings 8 weeks before independent shape classification and area measurements. Tendon shape was visually assessed on transverse proton density images and categorized as oval, general flat, flattened with medial convexity, or flattened with lateral convexity. Associations with split tear were evaluated using multivariable logistic regression.

**Results:**

Flat-shaped tendons were more common in the split tear group (91.6%) than in controls (82.1%), while oval tendons were less frequent (8.4% vs. 17.9%, *p* = 0.007). The flattened with lateral convexity shape was most strongly associated with split tear. In the multivariable analysis, flat shape (odds ratio [OR] = 2.26, *p* = 0.021), larger cross-sectional area (OR per mm^2^ = 1.04, *p* = 0.059), and older age (OR per year = 1.03, *p* < 0.001) are independently associated with split tear. No significant differences were observed between right and left ankles. Inter-rater agreement was substantial for shape (*κ* = 0.71, AC1 = 0.74) and excellent for area (intraclass correlation coefficient = 0.95).

**Conclusions:**

A flat-shaped peroneus brevis tendon, an increased cross-sectional area, and older age are associated with an increased likelihood of peroneus brevis split tears. These features may serve as anatomical imaging biomarkers for early risk identification.

**Supplementary Information:**

The online version contains supplementary material available at 10.1007/s00256-025-05032-y.

## Introduction

Ankle sprains account for approximately 16–40% of all sports-related injuries [1, 2]. The most commonly injured ligaments are the anterior talofibular ligament and the calcaneofibular ligament, which can lead to lateral ankle instability and predispose individuals to peroneus brevis (PB) tendon pathology, including split tears. Diagnosing PB split tears remains challenging, and the true incidence is likely underestimated [3–5]. The exact etiology and incidence of peroneus brevis split tears remain uncertain. While chronic lateral ankle instability is a recognized risk factor, most tears are thought to be degenerative, often related to repetitive microtrauma or anatomical crowding. This study explores tendon shape as a potential intrinsic factor contributing to tear development.

PB split tears most frequently occur near the tip of the lateral malleolus, where both the PB and peroneus longus (PL) tendons course through the retromalleolar groove in close proximity within the superior peroneal tunnel [6, 7]. These injuries are often chronic and associated with underlying peroneal tendon instability [8]. The PB is more commonly affected than the PL, but the exact pathogenesis remains unclear [4]. One prevailing theory suggests that, during forced dorsiflexion, the PB tendon may become compressed between the PL tendon and the lateral malleolus, leading to splitting and impaired healing [9].

Several anatomical variants have been proposed as potential predisposing factors for PB split tears, although their clinical significance remains uncertain [10, 11]. These include the presence of a peroneus quartus muscle, variations in retromalleolar groove morphology, accessory tendons, and hypertrophy of the peroneal tubercle [11]. Tendon-specific variants, such as a low-lying PB muscle belly, have been proposed as potential risk factors, though findings across studies remain mixed [12, 13].

Magnetic resonance imaging (MRI) and ultrasound are the primary modalities for diagnosing PB tears [14, 15]. On MRI, split tears may appear as altered tendon morphology or increased signal intensity. A characteristic chevron-shaped PB tendon wrapping around the PL on axial images has been proposed as an indicator of a tear [16]. However, while previous studies have primarily focused on variations in surrounding anatomy, the role of the tendon’s intrinsic geometry––such as its cross-sectional shape and area––has only been explored in smaller cohorts [17]. One such study proposed a potential association between a boomerang-shaped peroneus brevis tendon and split tears, but the limited sample size precluded formal statistical validation [17]. This gap is significant in light of biomechanical theories suggesting that a boomerang-shaped tendon may increase shear stress during motion, potentially predisposing the tendon to longitudinal splitting [9, 17]. It can be hypothesized that flatter tendon shapes may predispose the tendon to increased mechanical stress, potentially making them more susceptible to developing split tears. Clarifying this relationship could improve diagnostic accuracy and help identify individuals who are at higher risk. Although two prior studies have suggested an association between boomerang-, chevron-, or bisected tendon shapes and PB tears, these were limited by small sample sizes [12, 13, 17].

The present study addresses this knowledge gap by systematically evaluating peroneus brevis tendon shape, cross-sectional area, laterality, and patient age as factors associated with the likelihood of split tears on MRI in a large cohort.

## Aim

This study aims to determine whether the PB tendon shape, cross-sectional area, side, and patient age are associated with the presence of split tears on MRI.

## Material and methods

### Ethics

The study was approved by the National Ethical Authority (approval number 2024–07283-02).

### Study design

This retrospective cross-sectional study was designed and reported in accordance with the Strengthening the Reporting of Observational Studies in Epidemiology (STROBE) guidelines for cross-sectional research [18].

### Setting

MRI examinations obtained at our center between January 2018 and December 2024 were retrospectively re-evaluated. Data analysis was performed from January to March 2025.

### Participants

Patients with a visible PB split tear on MRI were consecutively assigned to the split tear group, while those without a split tear were consecutively included in the control group.

The inclusion criteria for both groups were patients aged 18 years or older at the time of imaging, and the availability of high-quality axial MRI sequences at the level of the lateral malleolus. For the split tear group, inclusion required the presence of a partial or complete PB split, defined as visible separation into two components on axial images. For the control group, inclusion required normal signal intensity of the peroneal tendons on proton density- and T2-weighted sequences, along with an intact superior peroneal retinaculum. To further ensure diagnostic clarity, we only included tendons in the control group that demonstrated completely normal signal intensity on all sequences. This criterion was applied to reduce the risk of including cases with subtle non-complete split tear.

**Fig. 1:**
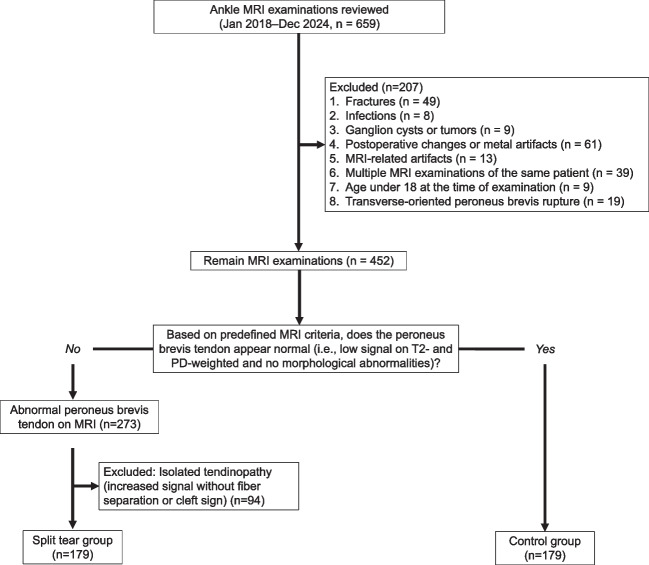
Flowchart illustrating the patient selection process. MRI examinations were reviewed consecutively in chronological order. Patients were prospectively assigned in parallel to the split tear or control group based on predefined MRI diagnostic criteria. Inclusion continued until 179 cases were reached in each group. Among the excluded cases, many had referrals citing lateral malleolar pain; these patients were excluded based on corresponding radiological findings, as specified in the general exclusion criteria. MRI = magnetic resonance imaging, PD = proton density weighted images

The exclusion criteria for both groups were postoperative changes in relation to lateral malleolus, metal-induced artifacts, tumors adjacent to the lateral malleolus, inflammatory conditions involving the peroneal tendons, and other pathologies such as bone marrow edema, prior lateral malleolar fractures, peroneal tendon dislocation and luxation, complete transverse tendon rupture, or laceration. We excluded patients with fibular tip fractures to minimize the influence of more severe traumatic injuries that may distort tendon morphology or surrounding structures. In the control group, we also excluded patients whose referrals indicated symptoms localized to the lateral malleolus. These cases are included among the excluded patients shown in the flowchart (Fig. 1), and their underlying findings are covered by the general exclusion criteria presented in the flowchart. A flowchart of the patient selection process is shown in Fig. 1.

### Participant characteristics

We reviewed 659 ankle MRI examinations and included 179 patients with a peroneus brevis split tear and 179 matched controls without a split tear (Fig. 1).

An overview of the demographic data for the study cohort is presented in Table 1. Patients with split tears had significantly higher weight and body mass index than patients without tears, while height did not differ significantly between the groups (Table 1).

**Table 1:** Demographic characteristics of patients with and without split tears

	Male	Female	Age (years)	Height (cm)	Weight (kg)	BMI (kg/m^2^)
Split tear	88 (49.2)	91 (50.8)	50.3 (18–81)	174.9 (134–198)	81.8 (51–144)	26.7 (17.5–44)
No split tear	93 (52.0)	86 (48.0)	42.2 (18–82)	176.0 (140–195)	76.7 (50–135)	24.8 (18.5–40)
*p* value	0.916	0.916	0.000	0.554	0.031	0.003

Data are presented as *n* (%) or median (range).

*BMI* body mass index, *n.s*. not significant.

In the control group, we also excluded patients whose referrals indicated symptoms localized to the lateral malleolus. These cases are included among the excluded patients shown in the flowchart, and their underlying findings are covered by the general exclusion criteria presented in Fig. 1.

### Variables

#### Outcomes

The two primary outcomes of this study were the morphological shape of the PB tendon, categorized as flattened with lateral convexity, flattened with medial convexity, generally flat, or oval (categorical variable); and the cross-sectional area of the PB tendon, measured in square millimeters (continuous variable).

#### Predictors

The main predictor variables analyzed were the shape of the PB tendon, as classified above, which may be associated with the occurrence of a split tear; laterality of the ankle (right or left); and age (in years).

### Data sources and measurements

#### MRI protocol

All MRI examinations were consecutively retrieved from the Picture Archiving and Communication System at our center. All MRI examinations were conducted using a dedicated ankle coil to maintain consistent joint positioning. Foam wedges were used within the coil to stabilize the ankle and minimize motion or positional shifts during imaging. Imaging parameters included a 14-cm field of view, voxel dimensions of 0.45 × 0.53 × 3.00 mm, and a slice thickness of 3 mm. The following imaging sequences were evaluated: T1-weighted imaging with an echo time of 11.5 ms (ms) and a repetition time of 700–750 ms, proton density-weighted turbo spin echo with an echo time of 45 ms and a repetition time of 2800–5000 ms, and T2-weighted turbo spin echo with an echo time of 60 ms and a repetition time of 3000–5000 ms.

#### Group assignment and image selection for evaluation

Two musculoskeletal radiologists independently assigned patients to the split tear or control group by reviewing the full MRI examination using predefined criteria eight weeks prior to the start of the study. For each case, the axial slice immediately proximal to the tear (or the corresponding level in controls) was selected and anonymized. Patients were assigned to the split tear group only if the peroneus brevis tendon demonstrated a clearly visible intratendinous cleft or separation into two distinct tendon fragments, consistent with a split tear. Discrepancies between the radiologists were resolved through consensus discussion. We selected the slice just proximal to the typical site of the split tear, as these tears most often occur at the apex of the lateral malleolus and propagate distally. This slice was then presented in random order to blinded raters, without any information about group assignment.

Observations from a preliminary review of 50 patients (25 with and 25 without split tears) showed that the cross-sectional shape of the PB tendon remained consistent along the short retromalleolar segment. Based on this finding, we selected a single standardized axial slice––immediately proximal to the tear or corresponding level in controls––for all shape assessments.

#### Tendon segmentation and shape analysis

Tendon segmentation was performed using a custom macro (Supplementary material 1) in ImageJ software (version 1.54 g). To assess intrarater repeatability of the macro, a musculoskeletal radiologist with 10 years of experience repeated the segmentations on 60 randomly selected images after a 4-week interval (see Supplementary material 2 for full validation details). Agreement between measurements was evaluated using the intraclass correlation coefficient (ICC[A,1]) and Bland–Altman analysis.

The PB tendon, PL tendon, and fibula were manually delineated by two independent raters: a medical student and a musculoskeletal radiologist with 10 years of experience. The macro-extracted *x*- and *y*-coordinates and automatically calculated the cross-sectional area of each segmented structure. Tendon morphology was visually evaluated and classified into one of four categories: generally flat, flattened with lateral convexity, flattened with medial convexity, or oval. The tendon shape classification is derived from the ratio of tendon thickness (shortest diameter) to width (longest diameter).

**Fig. 2:**
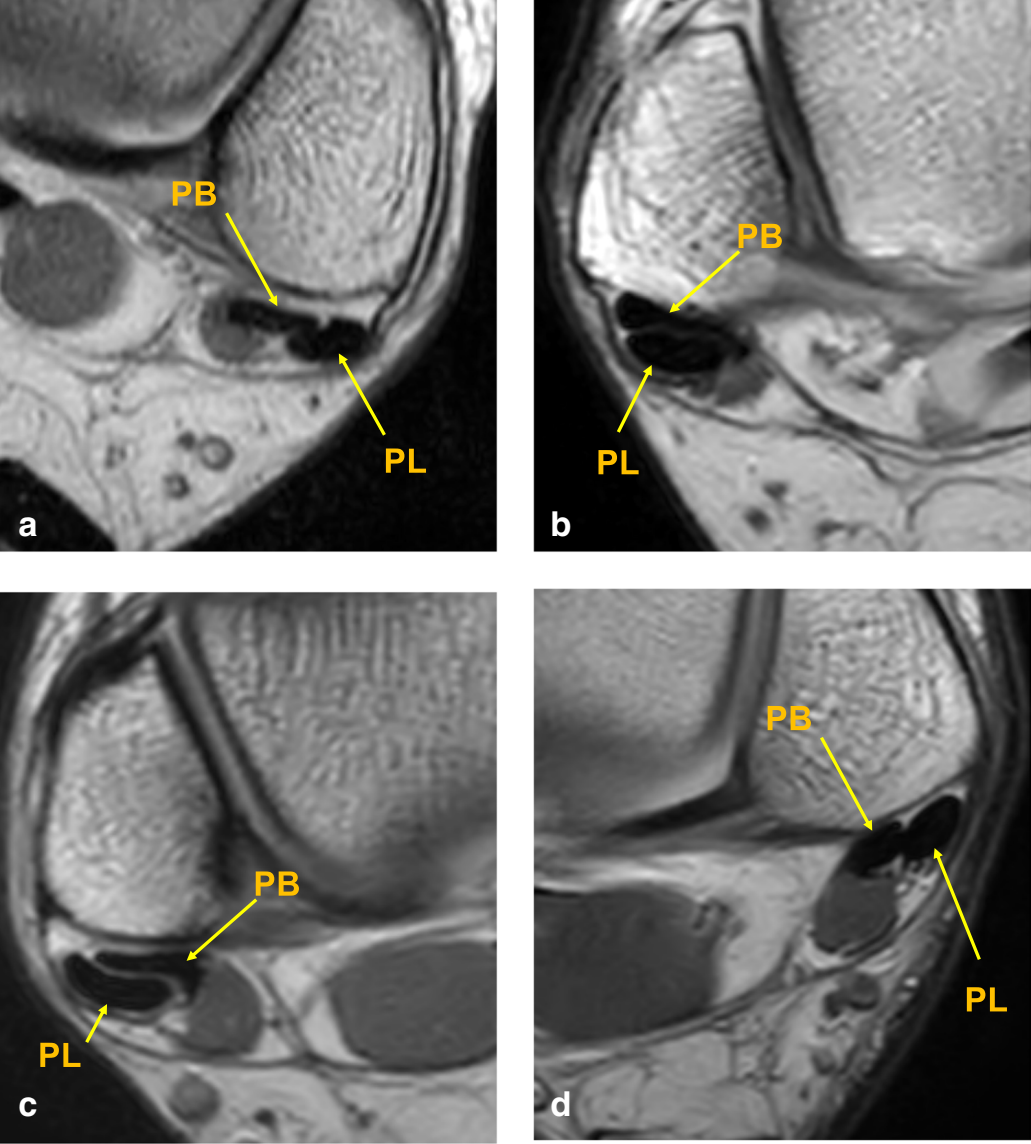
Axial proton density-weighted MR images (transverse sections) illustrating peroneus brevis (PB) and peroneus longus (PL) tendon morphology. **a** Generally flat shape, left ankle. **b** Flattened with lateral convexity, right ankle. **c** Flattened with medial convexity, right ankle; **d** Oval shape, left ankle. PB = peroneus brevis; PL = peroneus longus

The general flat configuration refers to a tendon with uniform thickness throughout, where the width is substantially greater than the thickness, giving it a flat appearance (Fig. 2). The flattened with lateral convexity variant also appears flattened but features a distinct outward bulge along the lateral border, resulting in increased thickness on the lateral side. By contrast, the flattened with medial convexity type presents a similar overall profile but with the convexity located medially, leading to greater thickness along the medial edge. The oval shape is characterized by a more rounded cross-section, in which the width slightly exceeds the thickness.

The tendon shape classification used in this study was developed by our research group and has demonstrated high inter-rater reliability in an independent validation study, currently accepted for publication.

We used self-reported height and weight values collected prior to the MRI examination, as these have been shown to correlate strongly with actual measurements and are considered valid for use in research studies [19].

### Raters

Two musculoskeletal radiologists (with 6 and 10 years of experience) independently selected the axial slice proximal to the split tear. Segmentation was performed by the same radiologists and a trained non-radiologist. Tendon shape was classified by consensus, while cross-sectional area was calculated as the mean of both raters’ values.

### Study size

Sample size estimation was based on a pilot analysis of 50 patients (25 with and 25 without split tears), which yielded a Cramér’s *V* of 0.186 for the association between tendon shape and tear status–consistent with a small-to-moderate effect. Using *α* = 0.05, power = 0.80, and 3° of freedom, the required sample size for a chi-square test was 332. A 10% buffer was added to account for exclusions, resulting in a final target of 358 patients (179 per group).

### Statistical methods

All statistical analyses were performed using RStudio (version 4.4.3).

Differences in the tendon shape distribution between groups were assessed using chi-square tests with Bonferroni correction for multiple comparisons. The cross-sectional area was compared using Welch’s *t*-test to account for unequal variances. To evaluate side-related differences in tendon shape, separate chi-square tests were performed for each category, with Bonferroni correction applied (adjusted *p* < 0.0125).

To identify PB tendon variables associated with split tears, we performed multivariable logistic regression with the presence of a split tear as the binary outcome. Predictors were pre-specified based on anatomical plausibility and prior literature: tendon shape (flat vs. oval), cross-sectional area (mm^2^), and patient age (years). All flattened types (generally flat, flattened with medial convexity, and flattened with lateral convexity) were grouped as “flat” and compared with the oval shape. All variables were entered simultaneously, without automated selection. We reported odds ratios (ORs), 95% confidence intervals (CIs), and *p* values.

Model discrimination was assessed using the area under the receiver operating characteristic curve, and calibration was evaluated both graphically and by comparing predicted and observed probabilities. Multicollinearity was examined using generalized variance inflation factors, with values of > 2 indicating concern.

To test robustness, we performed two sensitivity analyses: Firth logistic regression using the brglm2 package in R, and a reduced model excluding age. Predicted probabilities were generated for combinations of shape, area (10–25 mm^2^), and age (20–80 years).

#### Inter-rater reliability

To evaluate consistency in cross-sectional area measurements, the ICC(2,1) was used. Agreement in tendon shape classification was assessed using both Cohen’s Kappa and Gwet’s AC1 because Cohen’s Kappa is known to be susceptible to prevalence-related distortion, commonly referred to as the Kappa paradox [20].

## Results

### Association between anatomical tendon shape and split tears

The unweighted inter-rater agreement for tendon shape classification was substantial, according to Landis and Koch [21], with a Gwet’s AC1 of 0.741 and a Cohen’s Kappa of 0.710 (Supplementary material Table A1. Final shape assignments were determined by consensus between raters.

In the split tear group (*n* = 179), flat shapes were more prevalent, seen in 164 cases (91.6%), whereas the oval shape was present in only 15 cases (8.4%). In the group without a split tear (*n* = 179), flat PB tendon shapes were identified in 147 cases (82.1%), while the oval shape was observed in 32 cases (17.9%). This difference in distribution was statistically significant (Fisher’s exact test, *p* = 0.0073).

**Fig. 3:**
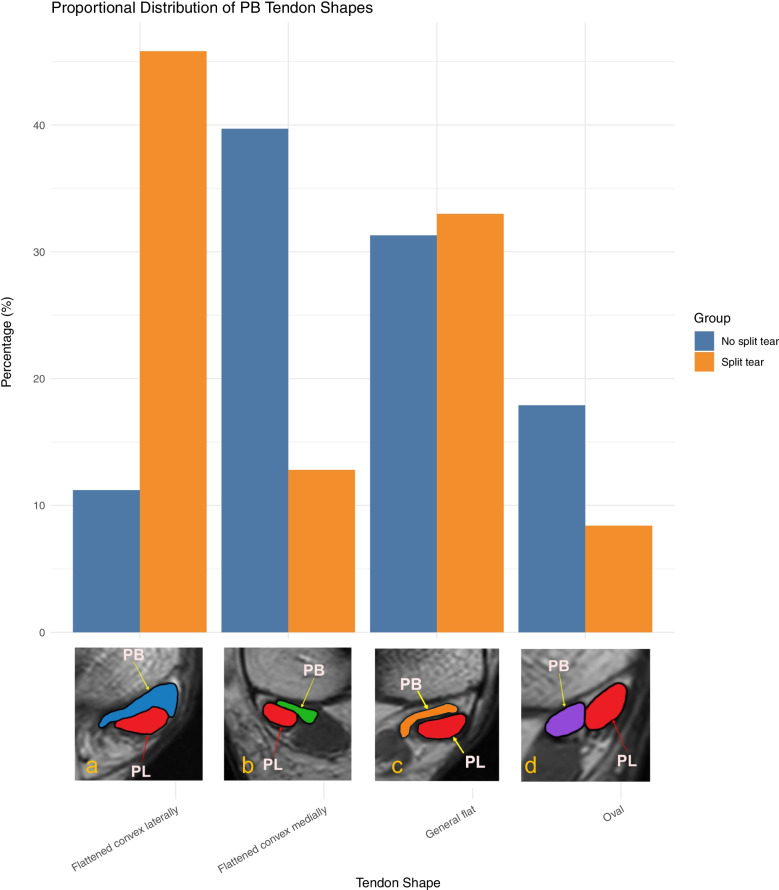
Distribution of PB tendon shape variants in patients with and without split tears. The bar plot shows the frequency of each tendon shape observed in both groups. PL––peroneus longus tendon, PB––peroneus brevis tendon. Proton density weighted images, left side: a, c, d; right side: b

The flattened with lateral convexity shape was the most common configuration among patients with split tears (Table 2, Fig. 3).

**Table 2:** Distribution of PB tendon shape variants among patients with and without split tears

Shape of PB	No split tear	Split tear
Flattened with lateral convexity	20 (11.2)	82 (45.8)
Flattened with medial convexity	71 (39.7)	23 (12.8)
Generally flat	56 (31.3)	59 (33.0)
Oval	32 (17.9)	15 (8.4)
Total	179 (100)	179 (100)

### Comparison of tendon shape distribution in split tear and control groups

Tendon shape was significantly associated with the presence of a split tear (*χ*^2^ = 51.03, *p* < 0.0001). Pairwise chi-square tests were used to identify which specific shape categories contributed to this difference (Table 3). To minimize the risk of false-positive findings due to multiple comparisons, a Bonferroni correction was applied. Post hoc pairwise tests revealed that the flattened with lateral convexity shape differed significantly from all other shapes, and the flattened with medial convexity shape differed from the generally flat shape (Table 3). No other comparisons remained significant after correction.

**Table 3:** Pairwise analysis of tendon shape categories

Shape 1	Shape 2	Raw *p* value	Bonferroni-corrected *p* value
Flattened with lateral convexity	Flattened with medial convexity	1.366 × 10^−14^	8.194 × 10^−14^
Flattened with lateral convexity	Generally flat	1.422 × 10^−5^	8.530 × 10^−5^
Flattened with lateral convexity	Oval	2.351 × 10^−8^	1.411 × 10^−7^
Flattened with medial convexity	Generally flat	1.389 × 10^−4^	0.001
Flattened with medial convexity	Oval	0.460	1
Generally flat	Oval	0.038	0.228

### Comparison of cross-sectional area between split tear and control groups

#### Validation of macro-based measurements

Macro-based measurements demonstrated excellent intrarater repeatability. The ICC for repeated area measurements was 0.996 (95% CI 0.993–0.998; *p* < 0.001). Bland–Altman analysis showed a mean difference of − 0.15 mm^2^, with limits of agreement ranging from − 1.05 to 0.76 mm^2^, indicating negligible bias (Supplementary material 2, Fig. A1). The Pearson correlation coefficient was 0.996 (95% CI 0.994–0.998; *p* < 0.001), and the median absolute difference between readings was 0.35 mm^2^.

#### Inter-rater reliability

Inter-rater reliability for cross-sectional area measurements was assessed using ICC(2,1) and interpreted according to Landis and Koch [21]. Agreement was classified as almost perfect, with ICCs of 0.92 for the fibula, 0.87 for the PL, and 0.95 for the PB. Final area values were averaged between raters to ensure consensus.

#### Group comparison

The PB tendon had a larger mean cross-sectional area in the split tear group (15.6 mm^2^) than in the non-tear group (14.1 mm^2^). Welch’s *t*-test confirmed this difference as statistically significant (*p* = 0.006; 95% CI 0.44–2.55 mm^2^).

### Comparison of tendon shape and cross-sectional area between sides

We evaluated whether PB tendon shape or cross-sectional area differed between sides. Four separate chi-square tests were performed for each tendon shape in patients with split tears, with Bonferroni correction applied (adjusted significance threshold *p* < 0.0125). No significant differences in shape distribution between sides remained after correction. For example, the flattened convex medially shape appeared in 37 left-sided and 57 right-sided cases (uncorrected *p* = 0.039; adjusted *p* = 0.157). The generally flat (64 left, 51 right; *p* = 0.225), flattened with lateral convexity (49 vs. 53; *p* = 0.692), and oval (28 vs. 19; *p* = 0.189) shapes also showed no significant side-to-side differences after adjustment.

A Wilcoxon rank-sum test comparing the PB cross-sectional area (mm^2^) between sides revealed no significant difference (*W* = 15,336, *p* = 0.49), with a negligible effect size (rank-biserial *r* = − 0.04). The median area was 14.0 mm^2^ (interquartile range 11.1–17.0) on the left and 13.9 mm^2^ (interquartile range 11.6–17.5) on the right.

Two-way analysis of variance showed a significant main effect of split tear status (*F*(1, 354) = 7.76, *p* = 0.01), confirming larger cross-sectional areas in tendons with split tears. No significant effect of side (*F*(1, 354) = 1.27, *p* = 0.26) or interaction between tear status and side (*F*(1, 354) = 0.12, *p* = 0.73) was observed (Supplementary material Table A2).

**Fig. 4:**
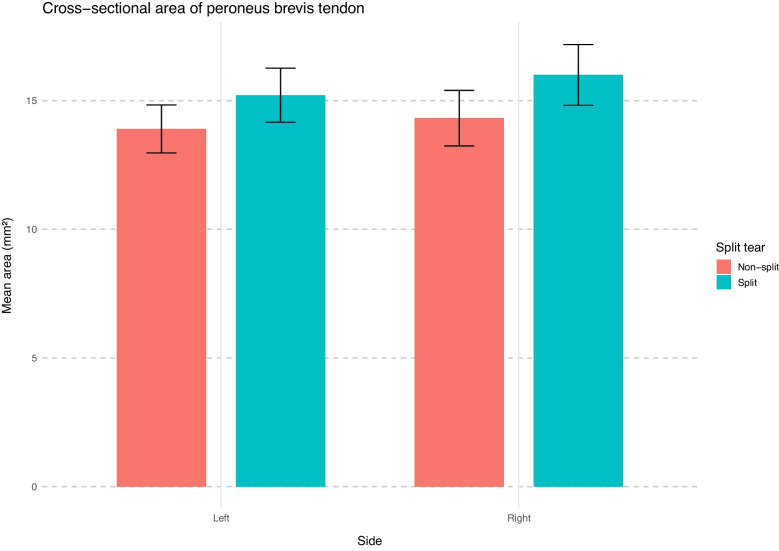
Mean cross-sectional area of the peroneus brevis tendon (mm^2^) by split tear status and side. Bars represent group means with 95% confidence intervals

Group means for cross-sectional area by split tear status and side are summarized in Supplementary material Table A3. The highest values were observed in the right-sided split tear group (Fig. 4).

### Variables associated with of split tear and likelihood stratification

**Fig. 5:**
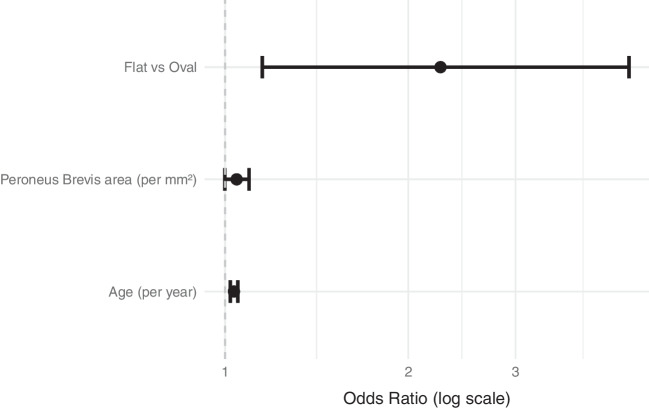
Adjusted odds ratios for variables associated with split tear. Forest plot displaying adjusted odds ratios with 95% confidence intervals from the multivariable logistic regression model. The *x*-axis is plotted on a logarithmic scale; the dashed vertical line represents the null value (odds ratio = 1)

We developed a multivariable logistic regression model to estimate the probability of PB split tears based on tendon morphology (flat vs. oval), cross-sectional area, and patient age. All three predictors were associated with an increased likelihood of a split tear (Table 4, Fig. 5). Flat-shaped tendons were significantly more likely to be associated with split tears than oval-shaped tendons (OR = 2.26, 95% CI 1.15–4.61, *p* = 0.021). The cross-sectional area of the PB tendon showed a borderline association with split tear (OR per mm^2^ = 1.04, 95% CI 0.999–1.09, *p* = 0.059). Age was independently associated with the likelihood of split tear, with each additional year increasing the odds of a split tear by approximately 3% (OR per year = 1.03, 95% CI 1.02–1.05, *p* < 0.001).

**Table 4:** Multivariable logistic regression: adjusted odds ratios and 95% CIs for the association between PB tendon shape, cross-sectional area, patient age, and presence of a split tear

Term	Estimate	Std. error	Statistic (*t*)	*p* value	95% CI (low)	95% CI (high)
(Intercept)	0.05	0.55	− 5.27	0.00	0.02	0.16
Flat shape	2.26	0.35	2.32	0.02	1.15	4.61
Cross-sectional area of PB	1.04	0.02	1.89	0.06	1.00	1.09
Age	1.03	0.01	4.67	0.00	1.02	1.05

*PB* peroneus brevis, *CI* 95% confidence interval, *Std*. *error* standard error. Estimates represent exponentiated coefficients (odds ratios)

**Fig. 6:**
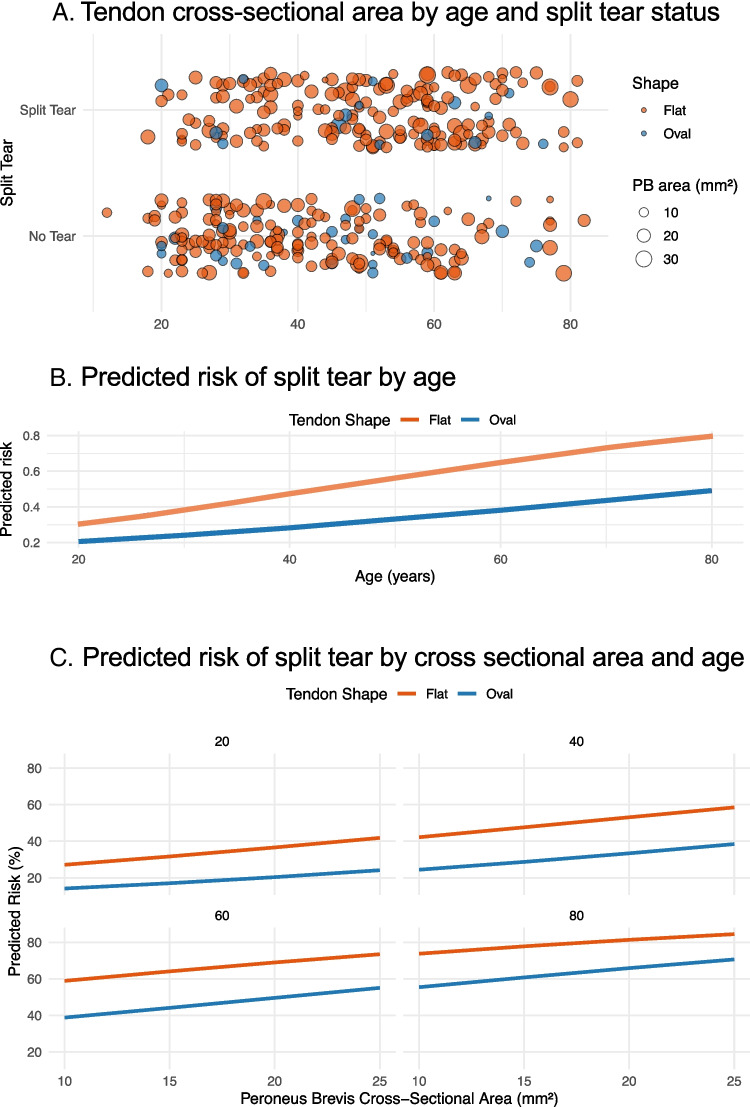
Predicted and observed relationships between tendon anatomy and split tear probability. **A** Individual tendons plotted by age and split tear status. Each circle represents one tendon, with size scaled to the peroneus brevis cross-sectional area and color indicating tendon shape (all flat shapes vs. oval). **B** Model-predicted split tear probability by age for flat and oval tendons, assuming a fixed median cross-sectional area. **C** Predicted probability as a function of both age and cross-sectional area, stratified by tendon shape

To support clinical interpretation, we calculated model-based predicted probabilities of split tears across representative anatomical and demographic profiles (Supplementary material Table A4, Fig. 6). The predicted likelihood of split tear increased progressively with both tendon area and age and was consistently higher in flat-shaped tendons. For example, in 80-year-old patients with a PB area of 25 mm^2^, the model estimated a probability of 84% for flat tendons compared with 71% for oval tendons. These differences were less pronounced in younger patients and in those with smaller tendon areas.

To assess the robustness and reliability of the regression model, we conducted multiple internal validations and sensitivity analyses (Supplementary material Tables A3–A6). Effect estimates remained stable across both a bias-reduced logistic regression model and a secondary model excluding age, supporting the consistency of the identified associations. Internal validation using bootstrapping yielded an area under the curve of 0.685, indicating moderate discriminatory ability (Supplementary material Fig. A2). Model calibration was satisfactory, with predicted and observed probabilities aligning closely across risk strata (Supplementary material Fig. A3). No concerning multicollinearity was detected; all predictors had generalized variance inflation factors below 2. A complete codebook detailing all variables, transformations, and model specifications is provided in the Supplementary material (Supplementary material Table A5).

## Discussion

The most important finding of this study is that flattened morphological variants of the peroneus brevis tendon are significantly associated with the presence of split tears. The flattened with lateral convexity shape showed the strongest association with split tears, while the oval shape was least associated. Moreover, the PB tendon demonstrated a larger cross-sectional area in the split tear group. The exact origin and incidence of peroneus brevis split tears remain unclear. While many are associated with lateral ankle sprains or instability, most appear to develop gradually due to chronic mechanical stress or anatomical predisposition. Degenerative changes are likely the dominant mechanism in many cases [22]. Our findings suggest that intrinsic tendon shape may be one such contributing factor.

The anatomical contour of the flattened with lateral convexity variant––characterized by a prominent lateral bulge––may predispose the tendon to localized compression or shearing between the PL tendon and the fibular groove, particularly during ankle movements. Thickening on the tendon’s lateral aspect may reduce its mobility during dorsiflexion, potentially leading to entrapment beneath the PL tendon––a mechanism previously proposed in earlier studies [23, 24]. The study by Ersöz et al. [17] identified a boomerang-shaped peroneus brevis tendon as associated with tear; however, their findings were based on a small cohort and a qualitative assessment of morphology. In contrast, our study employed a validated shape classification system in a statistically powered cohort using standardized 3-T MRI with a dedicated ankle coil.

The oval-shaped tendons were rarely associated with split tears, possibly due to reduced mechanical conflict. This may suggest that certain tendon shapes promote more favorable alignment or gliding within the superior peroneal tunnel. In our view, tendon shape is an underrecognized anatomical factor that may contribute to tendon tearing. Although a few reports in the literature mention tendon shape abnormalities, this issue has not been fully studied. Some authors have used terms such as “chevron” or “bifid” to describe morphological abnormalities seen in transverse cross-sections of PB tendons with split tears [12, 13].

The observation of a larger cross-sectional area in the split tear group aligns with previous studies of the Achilles and patellar tendons, where increased cross-sectional area has been linked to chronic mechanical loading, degenerative matrix changes, and elevated type III collagen deposition [25, 26]. While increased cross-sectional area is sometimes interpreted as a functional adaptation, our findings suggest that, in the context of PB split tears, it more likely reflects subclinical degeneration or chronic tendinopathy. Although we did not directly assess whether increased cross-sectional area alters PB shape, we assumed it does not, based on observations in the Achilles tendon [27], where thickening does not appear to result in substantial changes in cross-sectional shape, i.e., from oval to flat or vice versa. In our cohort, tendon shape and cross-sectional area appeared to provide complementary information. The flattened with lateral convexity shape was the most prevalent among individuals with split tears, and this group also exhibited the largest mean cross-sectional area. Together, these findings suggest a convergence of morphological risk factors, where both external geometry and internal structure may contribute to mechanical susceptibility. This may support a mechanical theory of split tear development; however, further studies are needed to evaluate this hypothesis. The observed area difference may partly reflect differences in body size, as patients with split tears had higher BMI and weight. However, the tendon area remained independently associated with split tears in multivariable analysis. We focused on anatomical imaging features, and future studies should explore how anthropometric factors influence tendon morphology and tear risk.

No significant differences in PB shape distribution or cross-sectional area were observed between the right and left ankles, suggesting that anatomical laterality has limited influence on PB morphology. The absence of side-related differences supports the notion that tendon shape is likely symmetrical [28] and not substantially affected by leg dominance or asymmetric loading. These findings indicate that PB tendon shape variants occur with similar frequency on both sides, suggesting that anatomical laterality is unlikely to play a major role in the development of split tears. Recent MRI-based analysis in individuals with normal peroneal tendons has confirmed the presence of flat-shaped variants as part of normal anatomical variability [29]. This supports the interpretation that tendon shape is an intrinsic anatomical feature, not merely a secondary effect of tear formation.

While intraoperative validation remains the gold standard in split tear diagnosis, it reveals only surgically treated cases and may introduce selection bias because not all peroneus brevis split tears are treated surgically. The current study aimed to reflect a broader clinical population evaluated on MRI. By applying defined diagnostic criteria and excluding equivocal findings, we sought to reduce misclassification. Our multireader approach improves group assignment reliability, as recent evidence shows that consensus MRI interpretation outperforms individual readings in diagnosing peroneus brevis tears [30].

To reduce potential bias from reactive or secondary morphological changes, patients with fractures were excluded. This approach enabled a more controlled evaluation of intrinsic tendon morphology as a potential anatomical risk factor of split tear. We consider this design choice essential to test whether flat-shaped tendons are inherently more likely to exhibit a split tear, independent of external trauma. We believe that the anatomical distribution of PB tendon shapes in patients with fibular fractures does not differ systematically from those without, and thus exclusion does not bias our shape-related findings. Rather, it enhances internal validity by reducing heterogeneity from severe or complex injuries that may otherwise obscure subtle associations between tendon shape and tear status. We acknowledge that the exclusion of patients with fractures and other complex pathologies may limit generalizability. However, this approach was chosen to reduce potential confounding and allow for a focused evaluation of intrinsic tendon morphology. We believe this design enhances the internal validity of our findings and provides a foundation for future studies in broader clinical populations.

To minimize bias, we excluded patients with prior fractures or superior peroneal retinaculum injuries, as these may alter tendon position or shape due to luxation or subluxation. Such changes could compromise reliable tendon classification. While this may limit generalizability to post-traumatic cases, it strengthens internal validity.

While the presence of a tear precludes the use of tendon shape as a predictive tool, our findings may have clinical value in at-risk populations––particularly athletes presenting with symptoms but no tear on imaging. In such cases, flat-shaped tendons may indicate increased susceptibility to injury, supporting early intervention and targeted rehabilitation. This concept is supported by similar applications of structural imaging biomarkers in tendon injury prevention, as recently demonstrated for the Achilles tendon [31].

The observed symmetry supports the idea that intrinsic tendon morphology, rather than lower limb dominance, may be more relevant in predisposing tendons to tear. While the dominant leg––most often the right––is more frequently used in activities such as kicking or stepping and is likely subject to greater use [28], our findings suggest that mechanical load is not the primary factor contributing to split tears. In this study, we found no differences in tendon shape related to side. This aligns with previous reports showing that although approximately 86% of individuals are right-leg dominant, such asymmetry does not appear to influence tendon morphology [32]. These findings further support the likelihood that PB tendon morphology is inherently symmetrical and unlikely to be shaped by leg dominance or uneven loading. Similar conclusions have been drawn for other tendons, including the Achilles and patellar tendons [33, 34]. While cross-sectional area may increase with repetitive loading over time, our data suggest that tendon shape is likely determined early in development and remains morphologically stable [35]. Although we did not assess limb dominance directly, future studies could help clarify whether long-term loading patterns influence tendon adaptation or contribute to split tear likelihood.

Cross-sectional area was significantly associated with split tear status. Tendons with a split tear exhibited a greater mean area than those without, independent of side. There was no significant main effect of side and no interaction between side and tear status, further suggesting that laterality does not influence tendon size in this context. Although cross-sectional area may increase in response to mechanical load, the enlargement observed in tendons with split tears may reflect pathological changes rather than physiological adaptation [35]. This interpretation aligns with previous research showing that tendinopathy and partial tears are often associated with localized thickening [36]. Whether increased tendon area precedes injury or results from degenerative change remains uncertain and should be explored in longitudinal studies.

Tendon morphology, cross-sectional area, and patient age were independently associated with split tear likelihood. Flat tendon shape emerged as the strongest determinant. Tendon deformation involves not only elongation but also sliding between fibers and fascicles. This internal sliding––particularly under shear and compressive forces––contributes to strain attenuation and fiber reorganization [37]. When mechanical load exceeds the tissue’s tolerance, such deformation may predispose the tendon to splitting. Cross-sectional area showed a borderline association, which may reflect morphological adaptation, chronic overload, or early degeneration [35].

Age contributed incrementally to likelihood, consistent with the progressive nature of tendinous degeneration and cumulative exposure to mechanical stress.

The model’s clinical utility lies in its ability to generate individualized, anatomy-based likelihood estimates using routinely available MRI features. These probabilities allow for stratification of patients into distinct risk groups, facilitating more informed interpretation of equivocal imaging findings. For example, in older patients with flat-shaped tendons, the model estimated a split tear likelihood exceeding 80%, whereas in younger patients with oval-shaped tendons, the likelihood was below 15%. Such probabilistic output may support preoperative decision-making, enhance radiologic accuracy, and contribute to more patient-centered care.

Model performance was acceptable, with satisfactory calibration across the full range of predicted probabilities. However, the development of PB split tears is likely influenced by a complex interplay of anatomical factors. Variants in adjacent structures––such as the peroneal groove or the presence of a peroneus quartus––may contribute to altered mechanical loading [38, 39]. Incorporating such anatomical features into future models may improve predictive performance.

PB split tear remains a challenging diagnosis, and any tool that improves diagnostic accuracy is valuable. Our findings suggest that PB tendon shape variants––particularly flattened with lateral convexity––may serve as early indicators of elevated likelihood, even in the absence of a visible tear. Although surgical confirmation remains the gold standard, MRI is the primary imaging tool used in clinical practice to diagnose peroneus brevis split tears. A recent multireader study demonstrated that MRI offers high diagnostic accuracy, particularly when examinations are reviewed in consensus [30]. In our study, we used a criterion for group assignment, requiring visible separation of the tendon into two distinct parts––a finding closely aligned with objective signs previously shown to improve diagnostic performance of MRI [40].

Tendon shape on MRI could help flag patients, such as athletes, for closer clinical monitoring, especially when symptoms are present. We believe our study contributes to expanding the role of MRI not only in detecting split tears but also in identifying tendon morphology associated with an increased likelihood of split tear.

This study demonstrates that flat shape and increased cross-sectional area of the PB tendon are associated with the presence of PB split tears. Oval-shaped tendons were less frequently observed in affected individuals. By contrast, no significant differences were found between the right and left sides in either tendon shape or cross-sectional area. Further, older age was associated with a higher occurrence of PB split tears. These findings suggest that intrinsic tendon morphology may help identify tendons at higher risk of split tear. These tendon-specific anatomical features may serve as valuable imaging biomarkers for identifying patients at increased risk and could support the future development of automated tools to enable earlier and more accurate diagnosis.

## Supplementary Information

Below is the link to the electronic supplementary material.

Supplementary file1 (DOCX 16.7 KB)

Supplementary file2 (DOCX 213 KB)

Supplementary file3 (DOCX 18.8 KB)

Supplementary file4 (PDF 5.86 KB)

Supplementary file5 (PDF 5.14 KB)

## Data Availability

The data supporting this case report are available from the corresponding author on reasonable request, in compliance with the Ethics Committee’s guidelines to ensure patient confidentiality.

## Abbreviations

CI: Confidence interval
ICC: Intraclass correlation coefficient
MRI: Magnetic resonance imaging
OR: Odds ratio
PB: Peroneus brevis
PL: Peroneus longus
