# Supplementary material for: Association between flat variants of the peroneus brevis tendon and split tears on magnetic resonance imaging
Source: Skeletal Radiol. 2025 Sep 13;55(2):263–76. doi: 10.1007/s00256-025-05032-y (PMC12743021; doi:10.1007/s00256-025-05032-y)
Supplement: Supplementary file 1 — Supplementary file1 (DOCX 16.7 KB) [file 256_2025_5032_MOESM1_ESM.docx]

# Supplementary material 1

// ==========================================

// ImageJ Macro: Manual Segmentation

// Author: Pawel Szaro

// Institution: University of Gothenburg, Sahlgrenska University Hospital

// Version: 1.2

// ==========================================

// === User-defined folder paths (update before use) ===

inputDir = "/path/to/your/dicom_folder"; // Folder containing DICOM files

outputFile = "/path/to/your/output/results.csv"; // Output CSV file with area measurements

coordinatesDir = "/path/to/your/output/coordinates"; // Folder to save TXT coordinate files

// === Create coordinates folder if it does not exist ===

if (!File.exists(coordinatesDir)) {

File.makeDirectory(coordinatesDir);

}

// === Initialize CSV output ===

File.delete(outputFile);

File.append("Filename,Fibula (mm^2),Peroneus Longus (mm^2),Peroneus Brevis (mm^2)\n", outputFile);

// === Process each DICOM file in the input folder ===

list = getFileList(inputDir);

for (i = 0; i < list.length; i++) {

if (endsWith(list[i], ".dcm") || endsWith(list[i], ".DCM")) {

open(inputDir + File.separator + list[i]);

// Extract base name for output naming

baseName = stripExtension(list[i]);

fileNameWithPrefix = "Name_" + baseName;

// === Check if pixel calibration is available ===

getPixelSize(unit, pixelWidth, pixelHeight);

if (pixelWidth == 1.0 && pixelHeight == 1.0) {

showMessage("Warning", "Pixel calibration not found for " + baseName + ". Measurements may be in pixels.");

}

// === Step 1: Segment Fibula ===

waitForUser("Manually outline the fibula on image: " + baseName + " and click OK.");

fibula_area = "NaN";

if (selectionType() != -1) {

run("Measure");

fibula_area = getResult("Area", nResults - 1);

saveCoordinatesToTXT("Fibula", fileNameWithPrefix, coordinatesDir);

}

// === Step 2: Segment Peroneus Longus ===

waitForUser("Manually outline the peroneus longus on image: " + baseName + " and click OK.");

longus_area = "NaN";

if (selectionType() != -1) {

run("Measure");

longus_area = getResult("Area", nResults - 1);

saveCoordinatesToTXT("Peroneus Longus", fileNameWithPrefix, coordinatesDir);

}

// === Step 3: Segment Peroneus Brevis ===

waitForUser("Manually outline the peroneus brevis on image: " + baseName + " and click OK.");

brevis_area = "NaN";

if (selectionType() != -1) {

run("Measure");

brevis_area = getResult("Area", nResults - 1);

saveCoordinatesToTXT("Peroneus Brevis", fileNameWithPrefix, coordinatesDir);

}

// === Save area measurements to CSV ===

File.append(fileNameWithPrefix + "," + fibula_area + "," + longus_area + "," + brevis_area + "\n", outputFile);

run("Clear Results");

close();

}

}

// === Function: Save selection coordinates to TXT file ===

function saveCoordinatesToTXT(structure, fileNameWithPrefix, coordinatesDir) {

getSelectionCoordinates(x, y);

output = "Coordinates for " + structure + " in image: " + fileNameWithPrefix + "\n";

if (x.length > 0) {

for (j = 0; j < x.length; j++) {

output += "Point " + (j + 1) + ": X=" + x[j] + ", Y=" + y[j] + "\n";

}

} else {

output += "No coordinates available.\n";

}

txtFile = coordinatesDir + File.separator + fileNameWithPrefix + "_" + structure + ".txt";

File.saveString(output, txtFile);

print("Coordinates saved to: " + txtFile);

}

// === Function: Remove file extension from filename ===

function stripExtension(fileName) {

dotIndex = lastIndexOf(fileName, ".");

return substring(fileName, 0, dotIndex);

}
