# Supplementary material for: Association between flat variants of the peroneus brevis tendon and split tears on magnetic resonance imaging
Source: Skeletal Radiol. 2025 Sep 13;55(2):263–76. doi: 10.1007/s00256-025-05032-y (PMC12743021; doi:10.1007/s00256-025-05032-y)
Supplement: Supplementary file 2 — Supplementary file2 (DOCX 213 KB) [file 256_2025_5032_MOESM2_ESM.docx]

## Supplementary material 2

**Macro validation details**

To validate the reliability of the custom ImageJ macro used for tendon segmentation and measurement, intra-rater repeatability was assessed. A board-certified musculoskeletal radiologist independently re-segmented 60 images after a four-week interval. The intraclass correlation coefficient (ICC[A,1]) for cross-sectional area measurements was 0.996 (95% confidence interval: 0.993–0.998), confirming excellent agreement. Bland–Altman analysis (Supplementary material Figure A1) showed a mean difference of –0.15 mm² between the two sets of measurements, with 95% limits of agreement from –1.05 to 0.76 mm². Pearson correlation was 0.996 (95% CI: 0.994–0.998; p < 0.001). The absolute difference between repeated measurements had a median of 0.35 mm² (range: 0.008–1.26 mm²). Coefficients of variation were 35.3% and 35.5% for the first and second measurements, respectively, reflecting expected dispersion due to the wide range of tendon sizes.


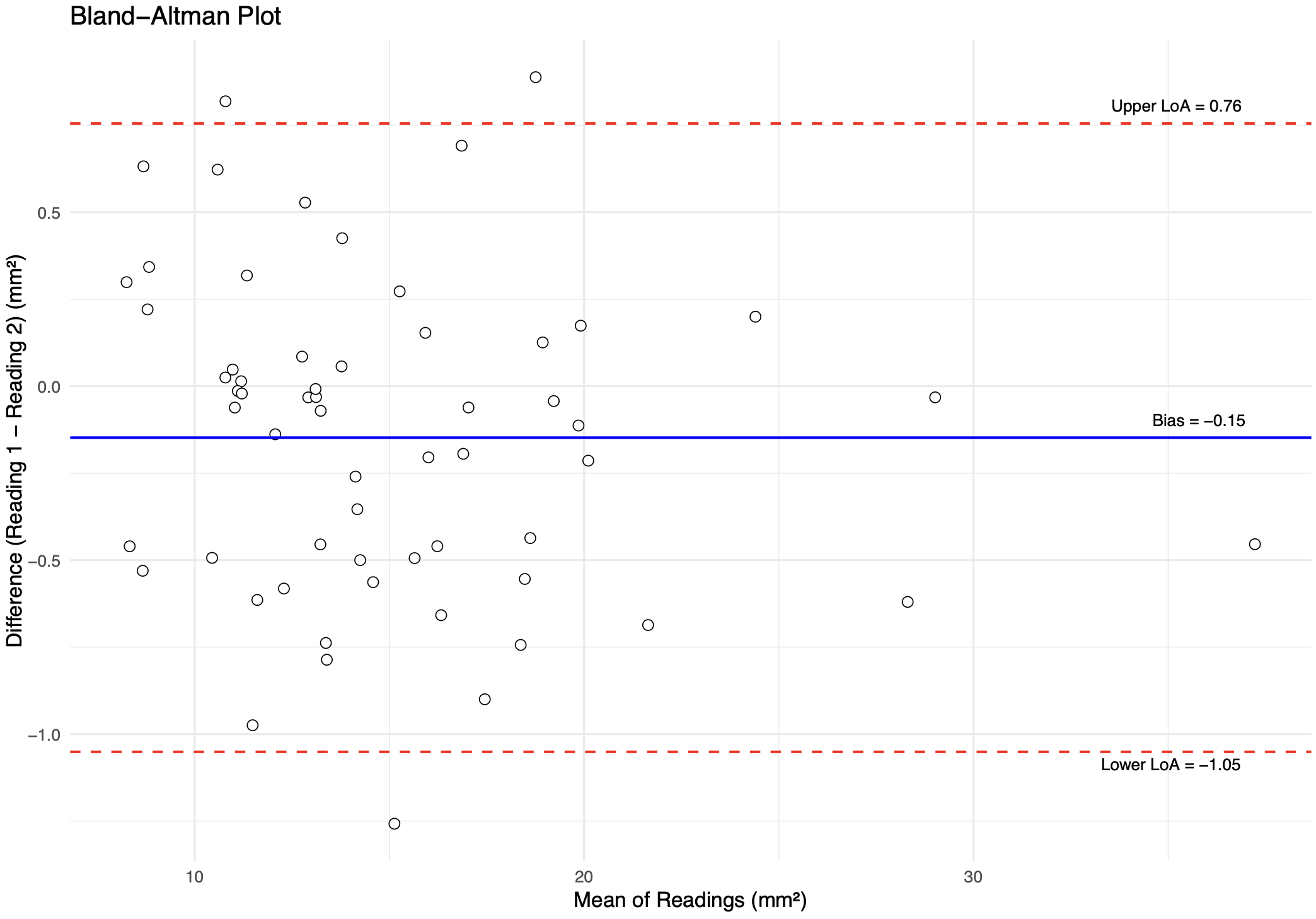


**Fig. A1.** Bland–Altman plot showing agreement between repeated macro-based area measurements. The mean difference was –0.15 mm² (solid blue line), with 95% limits of agreement ranging from –1.05 to 0.76 mm² (dashed red lines).
