# Supplementary material for: Association between flat variants of the peroneus brevis tendon and split tears on magnetic resonance imaging
Source: Skeletal Radiol. 2025 Sep 13;55(2):263–76. doi: 10.1007/s00256-025-05032-y (PMC12743021; doi:10.1007/s00256-025-05032-y)
Supplement: Supplementary file 3 — Supplementary file3 (DOCX 18.8 KB) [file 256_2025_5032_MOESM3_ESM.docx]

## Supplementary materials

## Tables

Table A1. Inter-rater agreement for peroneus brevis tendon shape classification, reported as Gwet’s AC1 and Cohen’s Kappa coefficients

|  | Coefficient | Standard error | 95% CI | p-value |
| --- | --- | --- | --- | --- |
| Gwet’s AC1 | 0.741 | 0.097 | (0.543 - 0.94) | <0.001 |
| Cohen’s Kappa | 0.710 | 0.110 | (0.503 - 0.92) | <0.001 |

CI, confidence interval

Table A2. Two-way analysis of variance assessing the effects of split tear status and side (left/right) on the cross-sectional area of the peroneus brevis tendon

| Factor | Df | Sum Sq | Mean Sq | F value | p-value |
| --- | --- | --- | --- | --- | --- |
| Split tear | 1 | 200.09 | 200.09 | 7.76 | 0.01 |
| Side | 1 | 32.79 | 32.79 | 1.27 | 0.26 |
| Split tear × Side | 1 | 3.02 | 3.02 | 0.12 | 0.73 |
| Residuals | 354 | 9134.1 | 25.8 | NA | NA |

Df, degrees of freedom; Sum Sq, sum of squares; Mean Sq, mean square; NA, not applicable

Table A3. Mean cross-sectional area of the peroneus brevis tendon (mm^2^), stratified by split tear status and side

|  | Side | Mean | SD | N | 95% CI (low) | 95% CI (high) |
| --- | --- | --- | --- | --- | --- | --- |
| No split tear | Left | 13.9 | 4.43 | 89 | 12.97 | 14.83 |
| No split tear | Right | 14.32 | 5.17 | 90 | 13.24 | 15.4 |
| Split tear | Left | 15.21 | 5 | 89 | 14.16 | 16.26 |
| Split tear | Right | 16 | 5.64 | 90 | 14.82 | 17.18 |

SD, standard deviation; N, sample size; CI, confidence interval

Table A4. Predicted probability of split tear across clinical profiles, based on model-estimated risk from combinations of tendon shape (all flat forms vs. oval), peroneus brevis cross-sectional area (mm^2^), and patient age (years)

| Age (years) | Area (mm^2^) | Shape | Predicted risk (%) |
| --- | --- | --- | --- |
| 20 | 10 | Oval | 14.1 |
| 20 | 10 | Flat | 27.1 |
| 20 | 25 | Oval | 24.1 |
| 20 | 25 | Flat | 41.8 |
| 40 | 10 | Oval | 24.4 |
| 40 | 10 | Flat | 42.2 |
| 40 | 25 | Oval | 38.4 |
| 40 | 25 | Flat | 58.5 |
| 60 | 10 | Oval | 38.8 |
| 60 | 10 | Flat | 58.9 |
| 60 | 25 | Oval | 55.1 |
| 60 | 25 | Flat | 73.5 |
| 80 | 10 | Oval | 55.4 |
| 80 | 10 | Flat | 73.7 |
| 80 | 25 | Oval | 70.6 |
| 80 | 25 | Flat | 84.4 |

Table A5. Variable Codebook. A complete description of all variables used in the regression model is provided, including coding, units, and transformations

| Variable | Description | Type | Coding |
| --- | --- | --- | --- |
| Split | Presence of split tear | Binary | 1 = Yes, 0 = No |
| Shape flat | Shape of PB tendon (flat vs. oval) | Binary | 1 = Any flat, 0 = Oval |
| Cross-sectional area PB | Cross-sectional area of peroneus brevis | Continuous | In mm² |
| Age | Patient age | Continuous | Years |
| Predicted | Predicted probability from model | Continuous | 0–1 |

PB – peroneus brevis.

Table A6. Collinearity diagnostics. Variance inflation factors (GVIFs) for all predictors were below conventional thresholds, indicating low multicollinearity and stable coefficient estimates

|  | vif(model) |
| --- | --- |
| Shape flat | 1.04 |
| Cross-sectional area PB | 1.03 |
| Age | 1.01 |
