# Supplementary figures and images for: Association between flat variants of the peroneus brevis tendon and split tears on magnetic resonance imaging
Source: Skeletal Radiol. 2025 Sep 13;55(2):263–76. doi: 10.1007/s00256-025-05032-y (PMC12743021; doi:10.1007/s00256-025-05032-y)

# ROC Curve

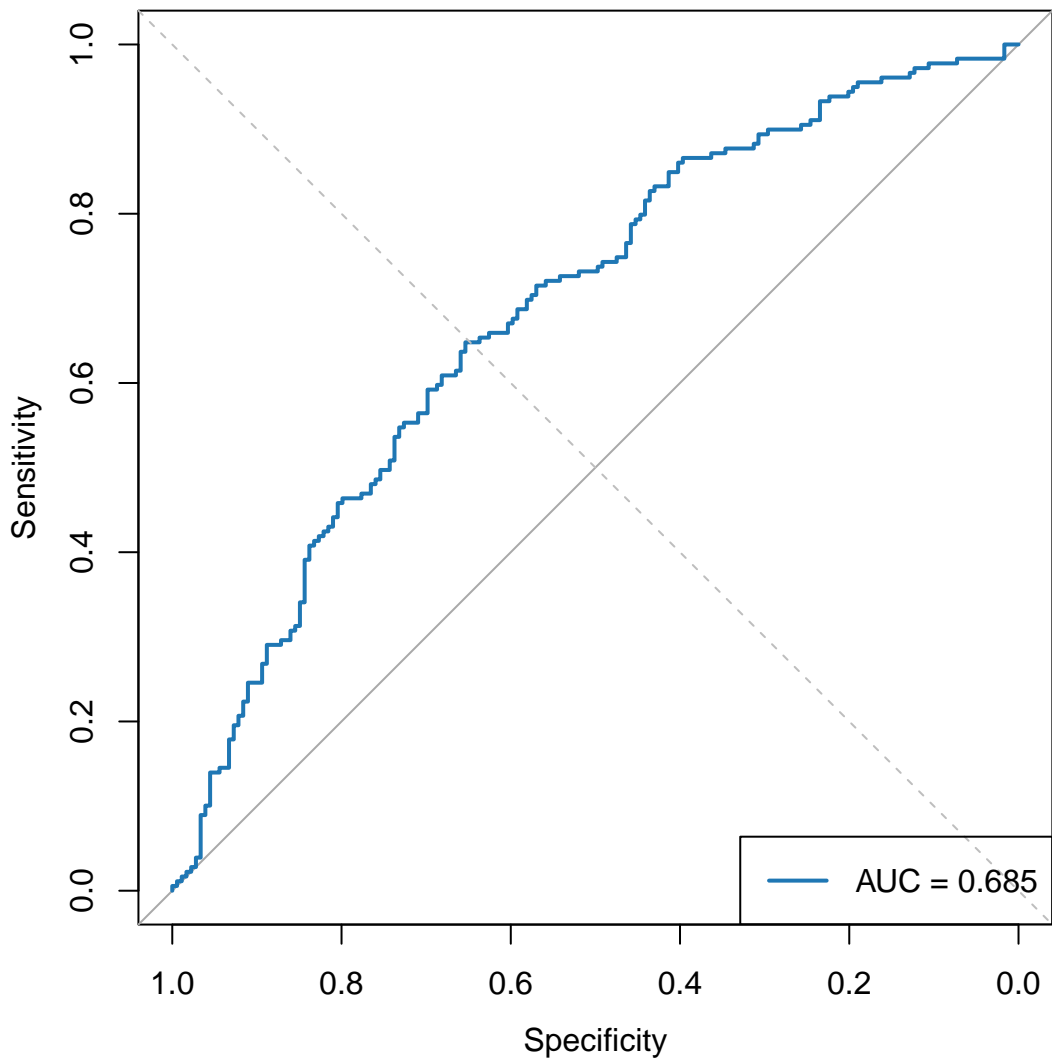

Supplement: Supplementary file 4 — Supplementary file4 (PDF 5.86 KB) [file 256_2025_5032_MOESM4_ESM.pdf]

# Calibration Plot

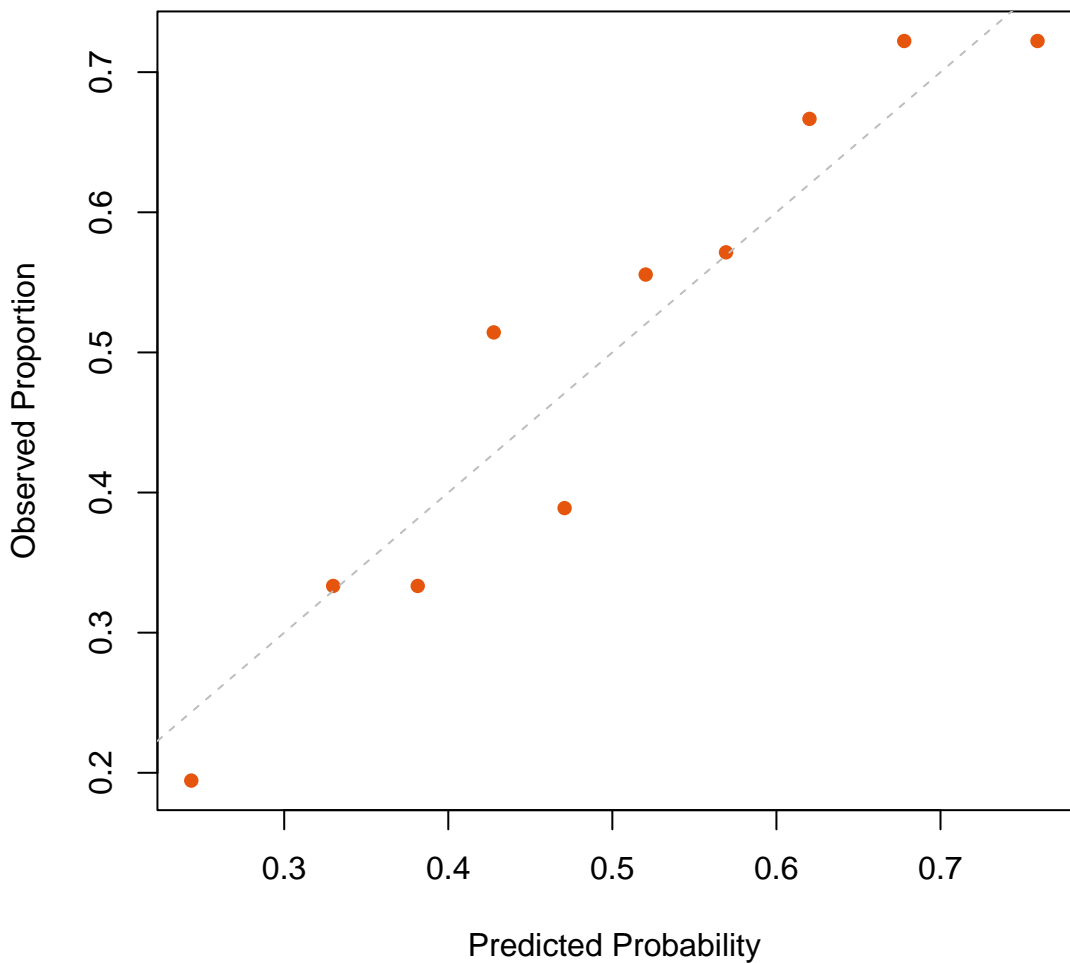

Supplement: Supplementary file 5 — Supplementary file5 (PDF 5.14 KB) [file 256_2025_5032_MOESM5_ESM.pdf]
